# Validation of immune complex dissociation methods for use with heartworm antigen tests

**DOI:** 10.1186/s13071-017-2442-8

**Published:** 2017-11-09

**Authors:** Melissa J. Beall, Andrea Arguello-Marin, Jan Drexel, Jiayou Liu, Ramaswamy Chandrashekar, A. Rick Alleman

**Affiliations:** 1 IDEXX Laboratories, Inc, One IDEXX Drive, Westbrook, ME 04092 USA; 2 Lighthouse Veterinary Consultants, Alachua, FL USA

**Keywords:** Antigen test, *Dirofilaria immitis*, Heartworm, Immune complex dissociation, Slow-kill

## Abstract

**Background:**

Antigen testing is routinely used to diagnose canine *Dirofilaria immitis* infections. Immune complex dissociation (ICD) methods, which were employed in the original heartworm antigen tests to release antigen that was bound by endogenous canine antibodies, were discontinued with improvements in assay reagents. The purpose of this study was to evaluate different ICD methods for detection of heartworm antigen by microtiter plate ELISA and assess the performance in samples from pet dogs.

**Methods:**

The original PetChek® Heartworm Test (IDEXX Laboratories, Inc.) utilized pepsin at an acidic pH for ICD prior to antigen testing. Performance and characteristics of the pepsin ICD method were compared with those for heat treatment (with and without EDTA) and acid treatment.

**Results:**

All four methods released complexed antigen in serum samples when tested using microtiter plate ELISA. Heat treatment required ≥600 μL of serum or plasma, whereas pepsin and acid methods needed only a 50-μL sample. Samples from 1115 dogs submitted to IDEXX Laboratories between 2014 and 2016 for investigation of discrepant heartworm results were evaluated with and without pepsin ICD using the PetChek Heartworm Test. Samples from 10% (*n* = 112) of the dogs were antigen positive with the ICD protocol only while 90% of the results remained unchanged. In a prospective study, antigen levels with and without ICD were evaluated for 12 dogs receiving pre-adulticide heartworm treatment with a macrocyclic lactone and doxycycline for 28 days. Serial samples revealed that three dogs had a reduction in detectable heartworm antigen within 4 weeks of initiating treatment. In these cases, heartworm antigen levels could be recovered with ICD.

**Conclusions:**

Heartworm antigen testing with ICD can be a valuable diagnostic tool for patients with discrepant results that have had intermittent use of a preventive, or have been treated with a macrocyclic lactone and doxycycline. Heartworm therapies may reduce antigen production and favor immune complexing in some dogs, resulting in false-negative results. Therefore, it is important to confirm positive heartworm antigen test results before initiating therapy.

## Background

**Fig. 1:**
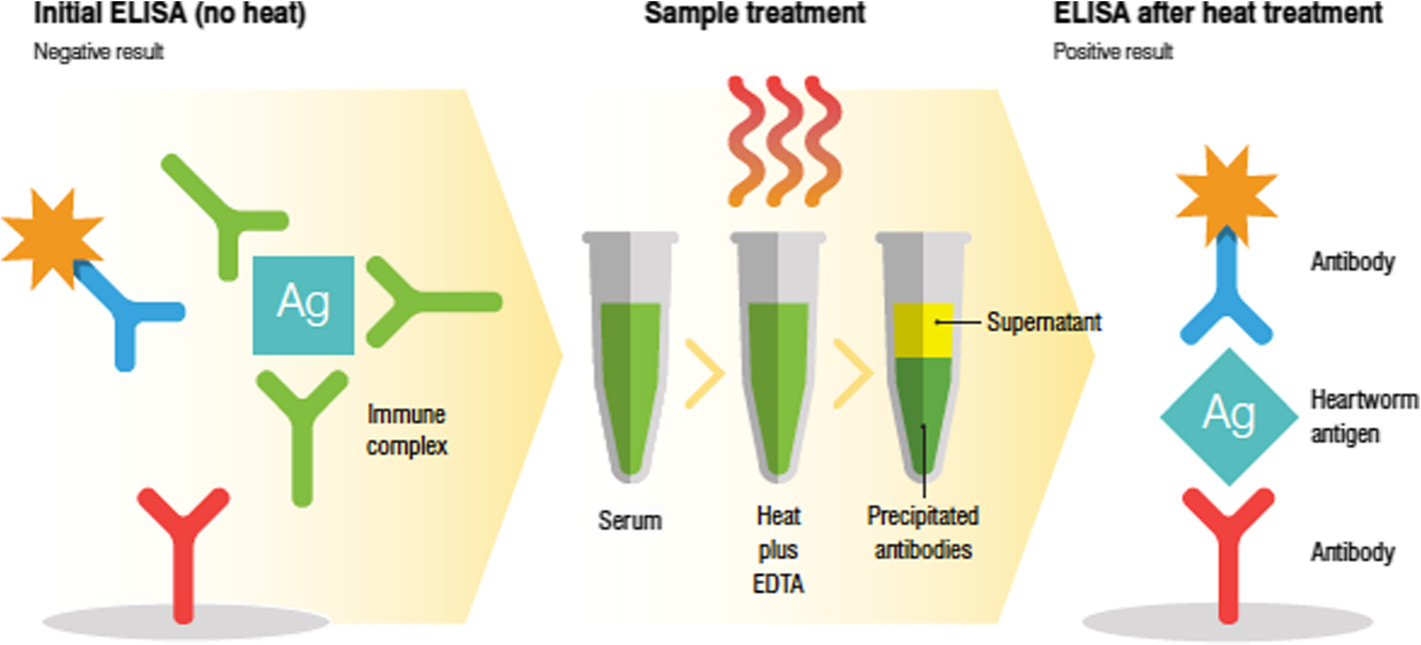
Illustration of antigen testing using an immune complex dissociation method, heat with EDTA, to liberate heartworm antigen

Diagnosis of *Dirofilaria immitis* infections in dogs is primarily accomplished by detection of circulating antigen in serum, plasma, or whole blood samples [1–3]. Parasite antigen may be present in the free state or may be trapped in immune complexes when antibodies are present in excess of antigen, leading to reduced sensitivity and false-negative test results. In early versions of the heartworm antigen test, immune complex dissociation (ICD) methods were used to free heartworm antigen from being bound to antibodies and improve the sensitivity of the test [4, 5]. Methods used for dissociating these antigen–antibody complexes generally included pretreatment of samples with heat and/or EDTA, which denatured immune complexes (Fig. 1). This step has been eliminated, however, from protocols of virtually all commercial *D. immitis* diagnostic assays over the past few decades as a result of improvements in assay technologies and antibody reagents [4].

Since early 2000, macrocyclic lactone heartworm preventives, often in combination with doxycycline, have been administered by veterinarians to heartworm-infected dogs to elicit a slow kill (SK) of heartworm adults when adulticide therapy is not an option for a particular dog [6]. Monthly preventive doses of macrocyclic lactones (with and without doxycycline) provide some level of efficacy in the death of heartworms; however, acceptable efficacy often requires extended periods of treatment, with some treatment durations as long as 1 to 2 years [7]. Heartworm disease may progress during the SK treatment regimen and can eventually lead to severe pulmonary damage with multiple pathologic changes [8]. Despite the presence of adult worms for some time throughout the lengthy SK treatment, antigen levels often decline due to a decrease in antigen production and the development of immune complexes, leading to false-negative antigen test results and inability to assess the status of infection in dogs receiving SK treatment [6, 7, 9]. Most experts agree that SK is not an acceptable method for treating patent heartworm infections and should only be considered when melarsomine is contraindicated [3, 10].

The purpose of studies described here were twofold: first, to evaluate different methods of ICD for the detection of heartworm antigen by microtiter plate ELISA, and second, to evaluate the use of ICD for antigen detection in samples from pet dogs, in particular for patients who have had discrepant results or have been treated with a macrocyclic lactone and doxycycline as a pre-adulticide therapy for a heartworm infection. The recommendation to use a combination of doxycycline and a macrocyclic lactone prior to the administration of melarsomine in *D. immitis–* infected dogs is based on clinical experience where it appears to reduce the likelihood and severity of adverse reactions [3, 10].

## Methods

### Evaluation of ICD methods

Several different techniques for ICD have been described in the literature and applied to the dissociation of heartworm antigen–antibody complexes. The original commercial PetChek® Heartworm Test (IDEXX Laboratories, Inc., Westbrook, Maine, USA) used a sample pretreatment method that included a pepsin reagent for ICD. In the current study, the pepsin method was compared with heat (with or without EDTA) and acidic pH methods. The methods for the ICD techniques evaluated in this study have been previously described [5, 11–14]. Briefly, the heat/EDTA method followed the procedure described by Weil et al., which diluted the serum sample with an equal volume of 0.1 M EDTA (pH 7.5) and heating to 100 °C for 5 min followed by centrifugation at 16,000 × *g*. Heat alone followed the method described by Little et al. and heated the sample at 104 °C for 10 min followed by centrifugation. Following ICD, all samples were tested on a microtiter plate ELISA (PetChek® Heartworm Test). Canine serum and plasma samples were used to evaluate the minimum starting sample volume needed to recover 100 μL of sample post-ICD for testing in the microtiter plate ELISA.

A pretreatment positive control (PPC) was developed to confirm that each method evaluated in this study dissociated the immune complexes and released heartworm antigen. The PPC, which consisted of purified heartworm antigen mixed with polyclonal antisera directed against heartworm antigen, was included each time an ICD protocol was performed. In addition to the PPC, samples from five heartworm-infected dogs, where one or two adult female heartworms had been recovered at necropsy, and one uninfected dog, with no adult heartworms recovered at necropsy, were used to compare the different ICD methods.

Of the ICD methods, the heat/EDTA technique was further evaluated using an additional 30 canine serum samples from dogs with adult heartworms at necropsy but having no or low levels of antigen when originally tested by the microtiter plate ELISA without ICD. To expand the population of samples without detectable heartworm antigen, canine serum samples from client-owned dogs (*N* = 181) that were submitted to a commercial reference laboratory for routine heartworm testing were evaluated. Heartworm antigen was not detected in these samples by the microtiter plate ELISA in the absence of ICD.

#### Retrospective analysis of field populations

To determine the frequency with which discrepant heartworm results are attributable to immune complexing of the antigen, heartworm test results from 1115 canine samples tested with and without the pepsin pretreatment method were analyzed. These samples had been submitted to IDEXX Laboratories between 2014 and 2016 for the investigation of discrepant heartworm test results. Specifically, results may have been inconsistent with clinical findings, or between different test methods (e.g., antigen vs microfilaria), or between different testing events (e.g., in-clinic vs reference laboratory).

### Prospective evaluation of HW infected dogs

Twelve dogs were recruited to evaluate the effect of administering a macrocyclic lactone and a 28-day course of doxycycline as pre-adulticide therapy on heartworm antigen levels. Private practices from Florida (*n* = 7) and Louisiana (*n* = 5) prospectively enrolled dogs with confirmed positive heartworm antigen test results and no history of having received heartworm prevention in the prior 4 months. Dogs were prescribed an appropriate dose of a commercially available macrocyclic lactone and doxycycline (5–10 mg/kg PO BID) from Day 0 to Day 28, prior to adulticide treatment. Serum samples were collected from the dogs on Days 0, 14, and 28 and tested for heartworm antigen before and after ICD treatment.

## Results

### Evaluation of ICD methods

All four ICD methods (heat/EDTA, pepsin, acidic, and heat) released heartworm antigen from immune complexes when the PPC was tested by microtiter plate ELISA. The methods required different minimum starting volumes of canine serum and plasma, however, to achieve the 100 μL sample volume required to perform the assay. A minimum starting sample volume of 100 μL was required for heat plus EDTA, 50 μL for pepsin or acidic, and 600 μL for heat (Table 1).

**Table 1:** Various methods of immune complex dissociation and the minimum starting volume of canine serum or plasma needed to recover 100 μL for the heartworm antigen ELISA test

Reference	Method	Minimum volume of sample required to recover 100 μL for testing	Reagents added to the sample
Weil [5]	Heat + EDTA	100 μL	100 μL 0.1 M EDTA
IDEXX PetChek® HW antigen test^a^	Pepsin	50 μL	35 μL S1 (acid) + 35 μL S2 (base)
Pokriefka [13] HIV antigen test	Acidic pH	50 μL	35 μL S1 (acid) + 35 μL S2 (base)
Little [11]	Heat	600 μL	NA

^a^The sample is added to an enzyme-coated well of the microtiter plate from the PetChek® HW antigen test kit. Solutions S1 and S2 are reagents contained within the PetChek® HW antigen test kit and are added sequentially before transferring the mixture to the plate assay

**Fig. 2:**
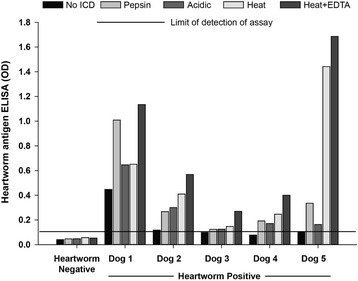
Comparison of immune complex dissociation methods for detection of heartworm antigen in samples from dogs with and without heartworm infections detected at necropsy

A comparison of the different ICD methods using samples from dogs with or without heartworm infections determined at necropsy is shown in Table 2. Heartworm antigen was not detected using any of the four ICD methods in the sample from the dog negative for heartworms at necropsy. The serum samples from five dogs with necropsy-confirmed heartworm infections had no detectable (*n* = 2) or low (*n* = 3) levels of heartworm antigen in the absence of pretreatment but all samples became antigen positive following ICD by the four methods (Fig. 2).

**Table 2:** Comparison of antigen detection with or without ICD using samples from necropsy-confirmed positive dogs and client-owned dogs

Dog population	Number of positive results				
	No ICD treatment	ICD treatment			
		Heat + EDTA	Pepsin	Acidic pH	Heat
Necropsy-confirmed positive (1–2 female worms); *n* = 5	3	5	5	5	5
Necropsy-confirmed positive (1–11 female worms); *n* = 30	17	29	ND	ND	ND
Client-owned (samples submitted to reference lab); *n* = 181	0	4	ND	ND	ND

*ICD* immune complex dissociation, *ND* not done

Based on required sample volume, performance, and simplicity of the method, the heat/EDTA technique was further evaluated using a larger population of canine samples. Of the 30 samples from dogs with heartworms at necropsy (but with no or low antigen levels when originally tested), 17 samples that were originally positive retested as positive for heartworm antigen using the heat/EDTA method. Twelve samples that were originally negative for antigen became positive for heartworm antigen using the heat/EDTA method. One sample remained negative for heartworm antigen despite ICD pretreatment (Table 2). Of the 181 canine serum samples from client-owned dogs that were negative for heartworm antigen without ICD, 177 (98%) remained negative following ICD. Four (2%) samples converted from negative to positive following pretreatment of the sample with heat/EDTA.

### Retrospective analysis of field populations

To evaluate the frequency with which discrepant heartworm antigen test results are affected by immune complexing, results from 1115 samples submitted between 2014 and 2016 for the investigation of discrepant heartworm results were analyzed. Most samples (1003; 90%) produced the same result with and without the pepsin method of ICD. Specifically, 772 (69%) samples remained negative for heartworm antigen and 231 samples remained positive for heartworm antigen with and without ICD. Samples from 112 dogs (10%) negative for heartworm antigen without ICD tested positive following treatment with the pepsin ICD method.

### Prospective evaluation of heartworm-infected dogs

**Fig. 3:**
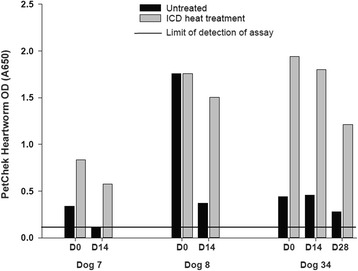
Heartworm antigen levels in serum samples from three dogs with low levels of antigen at diagnosis, shown with and without immune complex dissociation

Of the 12 dogs enrolled with positive heartworm antigen results, serum samples from 9 dogs had high antigen levels (high ELISA OD) when tested with and without ICD at all time points (data not shown). Three dogs, however, demonstrated a measurable decrease in antigen levels while receiving pre-adulticide therapy (Fig. 3). When Day 14 and Day 28 samples from these three dogs were tested without ICD treatment, the results were near the cut-off of the microtiter plate ELISA for heartworm antigen. These samples demonstrated enhanced detection of heartworm antigen following ICD treatment (Fig. 3).

## Discussion

Our study demonstrates that multiple methods are capable of dissociating circulating immune complexes containing heartworm antigen. The existence of immune complexes in heartworm-infected dogs has been known for some time [5, 15–17]. Diagnosis of other infectious diseases besides heartworm can have interference from immune complexes, such as the detection of p24 in HIV, circulating antigens in leishmaniasis, dengue, histoplasmosis, and others [13, 18–22]. Our study also demonstrates that the level of heartworm antigen detection by microtiter plate ELISA following ICD can vary by sample and by ICD method. A standardized ICD procedure is important to ensure the validity of the test results. A critical component of standardization is the inclusion of a pretreatment positive control, which confirms that the ICD method has been performed correctly each time a sample is tested. These standardized procedures and controls are not typically performed in-clinic but should be a part of reference laboratory protocols.

A number of recent reports indicate that the presence of heartworm antigen–antibody immune complexes can interfere with antigen detection [6, 11, 14]. In one study, archived serum samples from dogs in animal shelters (*N* = 165) that were negative for heartworm antigen were subsequently retested after heat treatment [14]. Following heat treatment, 11/154 samples (7.1%) became positive for heartworm antigen. This population primarily included dogs from an endemic area that were not receiving monthly heartworm prevention or other routine veterinary care. In our study, 10% of samples evaluated for discrepant heartworm test results demonstrated evidence of immune complexing that interfered with heartworm antigen detection. The full history on these cases was not available to document previous treatment or preventive use in these dogs. The frequency of false-negative heartworm test results due to immune complexes in both instances was higher than the 2% identified in the 181 serum samples from client-owned dogs reported in this study. Likewise, a recent study has demonstrated that dogs visiting a wellness care veterinary program had no false-negative test results for heartworm on their point-of-care screening tests due to immune complexes [23]. These results suggest that the frequency of immune complex–associated interference in heartworm test results is low in the population of well-cared-for-dogs but may be of concern in dogs with certain risk factors including inconsistent use of heartworm preventive products, administration of pre-adulticide type therapy or SK, and conditions associated with chronic immune stimulation. Therefore, methods of ICD are not appropriate for routine use in heartworm screening but may be valuable in cases in which heartworm infection is suspected and negative heartworm antigen results are obtained.

Several leading veterinary specialty organizations like the American Heartworm Society, the Companion Animal Parasite Council, and the European Scientific Counsel Companion Animal Parasites discourage the use of SK, citing compliance failure, potential for selecting for resistance, and the persistence of worms in the pulmonary arteries and eventual development of pulmonary pathology during the SK treatment [3, 10, 24]. Nevertheless, some veterinarians use the SK treatment for certain dogs, particularly those owned by clients who cannot afford adulticide treatment or when the medical risk is high. Treatment of heartworm-infected dogs with a macrocyclic lactone at preventative dosages with or without doxycycline also may reduce free heartworm antigen in some dogs. McCall et al. reported that dogs with experimentally induced infections of *D. immitis* treated with 16 monthly preventive doses of ivermectin and pyrantel pamoate showed declining levels of antigen during the study. The dogs in that study had live worms at necropsy, suggesting that the SK treatment interfered with either antigen production by the surviving worms or with the detection of complexed antigen [2]. Using a commercial microtiter well-based assay, Drake et al. reported that 15 dogs treated monthly with a macrocyclic lactone and doxycycline became antigen negative within 24 months [6]. Of the 15 dogs that were antigen negative by the initial assay, eight were found to be antigen positive following heating of the sample before re-testing. Therefore, dogs on SK treatment may be suitable candidates for ICD testing.

The combined administration of a macrocyclic lactone and doxycycline has been shown to have both adulticidal and microfilaricidal effects [9]. Female worms recovered from dogs receiving this therapy demonstrated significant inhibition of oogenesis/embryogenesis of filariae and had a loss of uterine content. Likewise, dogs in this treatment group demonstrated a reduction in circulating heartworm antigen. Decreased antigen production by the female parasite and the potential for immune stimulation as parasites die may shift the ratio of antigen to antibody favoring antigen bound in immune complexes. Hence, there is a risk of false-negative test results after initiating therapy with a combination of a macrocyclic lactone and doxycycline. Therefore, it is important to confirm positive heartworm antigen test results by another testing protocol before initiating any type of therapy.

## Conclusions

This study confirmed that multiple methods are effective for the dissociation of heartworm antigen from immune complexes. Some variability is evident across samples and methods, highlighting the importance of standardized protocols and controls to ensure reliable results. The heat/EDTA method as originally described by Weil et al. [5] is recommended due to ease of use, reliable performance with small sample volumes, and availability of reagents. Immune complexes were found to infrequently interfere with the performance of heartworm antigen tests in well-cared-for client-owned dogs. Therefore, ICD methods are not necessary for routine screening but should be considered when heartworm infection is suspected and antigen results are negative. This could also include dogs on SK or with chronic inflammation where antibody levels may exceed antigen concentration resulting in immune complex formation [6, 14]. Finally, this study identified a potential risk of false-negative test results in some dogs after initiating therapy with a combination of a macrocyclic lactone due to a reduction in detectable heartworm antigen levels that could only be recovered following ICD protocols.

## Abbreviations

ICD: Immune complex dissociation
PPC: Pretreatment positive control
SK: Slow kill
